# Polymicrobial Septic Peritonitis Caused by *Enterococcus faecium* and *Enterococcus casseliflavus* following Uterine Rupture in a Goat

**DOI:** 10.3390/vetsci11060268

**Published:** 2024-06-12

**Authors:** Gabriel S. dos Santos, Giovanna S. Francischetti, Natália F. Garritano, Stefano C. F. Hagen, Artur F. Cagnim, José Luiz Catão-Dias, José S. Ferreira Neto, Maria Claudia A. Sucupira, Marcos B. Heinemann

**Affiliations:** 1 Departamento de Medicina Veterinária Preventiva e Saúde Animal, Faculdade de Medicina Veterinária e Zootecnia, Universidade de São Paulo, São Paulo 05508-270, Brazil; gabriel.siqueira.santos@alumni.usp.br (G.S.d.S.);; 2 Departamento de Clínica Médica, Faculdade de Medicina Veterinária e Zootecnia, Universidade de São Paulo, São Paulo 05508-270, Brazil; 3 Departamento de Cirurgia, Faculdade de Medicina Veterinária e Zootecnia, Universidade de São Paulo, São Paulo 05508-270, Brazil; 4 Departamento de Patologia, Faculdade de Medicina Veterinária e Zootecnia, Universidade de São Paulo, São Paulo 05508-270, Brazil

**Keywords:** infectious peritonitis, uterine tears, dystocia, sepsis, enterococci

## Abstract

**Simple Summary:**

A pregnant miniature goat underwent an emergency cesarean section after calving one dead goatling. In the postoperative period, the goat had septic peritonitis caused by *Enterococcus faecium* and *Enterococcus casseliflavus*, exhibiting differing antimicrobial resistance profiles. Despite laparohysterectomy and abdominal lavage, euthanasia was required due to adhesions and necrotic lesions. A post-mortem examination revealed fibrino-necrotic septic peritonitis resulting from uterine rupture.

**Abstract:**

A one-year-old female miniature goat was presented to an emergency service after calving a dead goatling. Physical and ultrasonographic examination revealed the presence of a viable fetus; therefore, the goat was submitted to an emergency cesarean section. In the postoperative period, the animal had septic peritonitis caused by *Enterococcus faecium* and *Enterococcus casseliflavus*. Both bacterial strains showed contrasting antimicrobial resistance profiles. Laparohysterectomy and abdominal cavity lavage were performed, but, once the animal had adhesions and necrotic lesions in abdominal organs, euthanasia was executed. A post-mortem examination revealed fibrino-necrotic septic peritonitis secondary to uterine rupture. To the authors’ knowledge, this is the first detailed report of polymicrobial septic peritonitis in a miniature goat and the first report of septic peritonitis caused by *E. faecium* and *E. casseliflavus*.

## 1. Introduction

Uterine rupture is a peripartum reproductive emergence in domestic animals and a frequent consequence of dystocia in small ruminants [1,2]. It is a surgical emergence and is related to lower survival rates after birth due to possible complications [1].

Once the females’ reproductive tract is a non-sterile site [3,4], uterine rupture, mainly in septic metritis cases, possibly leads to septic peritonitis and, consequently, to sepsis [5,6]. Clinically important bacteria such as *Escherichia coli*, *Streptococcus* spp., and *Enterococcus* spp. are frequently isolated from a female’s reproductive tract [3,4] and traumatic lesions in this site can eventually spread them in the abdominal cavity. Besides that, *Enterococcus* spp.—Gram-positive, catalase-negative, and anaerobic facultative cocci—are intrinsically resistant to several antimicrobials [7], and it can impair pharmacological treatments of an infectious process before the proper microbiological diagnosis and antimicrobial susceptibility testing [8]. 

This study aimed to investigate the clinical, microbiologic, and anatomopathological findings of infectious peritonitis caused by *E. faecium* and *E. casseliflavus* in a miniature goat following a uterine rupture. There is a lack of information about septic peritonitis in domestic animals and no reports of polymicrobial peritonitis in small ruminants. 

## 2. Case Description

A one-year-old female miniature goat weighing 27.4 kg and with no history of abortion attended the Veterinary Hospital of the School of Veterinary Medicine and Animal Science from the University of São Paulo as an urgent referral. After approximately 120 days of pregnancy, the goat calved one dead goatling and, on the same day, the owners reported that the animal was prostrated and showed uterine contractions every five minutes. The goat was neither vaccinated nor dewormed and was fed with hay, corn, goat feed, and, occasionally, beetroot leaves.

Upon presentation (Day 1), the animal was tachycardic (156 bpm) and tachypneic (160 mpm). Abdominal and transvaginal palpation revealed the presence of another fetus, closed cervix, and purulent and fetid discharge from the vulva. While pending further investigations, supportive care was initiated, consisting of intramuscular (IM) dipyrone (25 mg/kg/8 h) and a single dose of IM gentamicin (3.3 mg/kg).

Hematology on Day 1 showed neutrophilia with a left shift, a decreased MCV and MCH, increased red blood cell and thrombocyte values, but normal hemoglobin and hematocrit values (Table 1). Serum biochemistry showed high creatinine and urea values and moderate hypoproteinemia and hypoalbuminemia (Table 1).

Once further ultrasonographic examination confirmed the presence of a viable fetus by measuring the heartbeat, the goat was submitted to an emergency cesarean section. During the surgery, the uterus was fetid and inflamed; thus, as post-surgical medications to fight infection, IM ceftiofur (2.2 mg/kg/24 h) and enrofloxacin (5 mg/kg/24 h) were prescribed, and, for pain and inflammation management, morphine (0.1 mg/kg/4 h), dipyrone (25 mg/kg/8 h), and meloxicam (0.5 mg/kg/24 h) were prescribed.

After the surgery, the goat was lethargic and showed a slight increase in temperature (39.1 °C) and red mucous membranes. It eliminated the placenta on the same day of the surgery (Day 1) and continued to have purulent discharge from the vulva. The goatling was born without deciduous incisive teeth and had incomplete hooves' development. It was prostrated and did not want to suckle colostrum, and a physical examination revealed pulmonary crackling. Even if nutritional support with esophageal probing were performed, on the next day, the goatling was more protracted and hypothermic (33 °C), had acidemia (pH: 7.17), and continued to have crackling pulmonary auscultation. Despite attempts to reverse the worsening of the animal’s health status, it had respiratory arrest, cardiac fibrillation, and died.

During the next seven days (Day 1 to Day 8), the goat was hyporexic, and abdominal palpation and percussion revealed abdominal sensitivity and that there was gas in the surgery region. Once hematology revealed neutrophilia with a left shift from Day 7 (Table 1), ceftiofur and enrofloxacin administration was suspended and IM penicillin (40,000 UI/24 h) was prescribed.

Ultrasonographic examination was performed (Day 9) and uterus alterations such as thickened and irregular walls, a presence of intramural gas, and marked interspersed gas content within the uterine structure were identified. Additionally, increased peritoneal echogenicity and free fluid in the abdominal cavity with increased echogenicity and cellularity were observed. A puncture was aseptically performed using a catheter and syringe, obtaining 400 mL of dark green fetid liquid content.

The fluid was streaked onto blood agar 5% and incubated at 37 °C overnight. Two morphologically distinct gamma-hemolytic bacteria colonies were obtained and bacterial identification with matrix-assisted laser desorption/ionization time-of-flight (MALDI-TOF) identified them as *E. faecium* and *E. casseliflavus*. The antimicrobial susceptibility profile was assessed with a disk-diffusion test according to the Clinical and Laboratory Standards Institute (CLSI) protocol [7]. Both bacteria showed contrasting antimicrobial resistance profiles. They were resistant to penicillin, ampicillin, and tetracycline and had intermediate resistance to erythromycin, chloramphenicol, and vancomycin (Table 2), resulting in challenges for the treatment of peritonitis using antimicrobials.

It was decided to perform a laparotomy aiming for ovarian-salpingo-hysterectomy and abdominal cavity lavage. Due to progressive anemia since Day 9 and once the hematocrit value was 0.11 (11%) (Table 1), it was necessary to have a blood transfusion from another goat with approximately 1100 mL divided into two blood transfusion sections on two different days (Day 12 and Day 14).

During the surgical procedure, uterine rupture and free seropurulent fluid in the abdominal cavity were identified. A laparohysterectomy and cavity lavage were performed but, once there was adhesion in the abdominal organs and the omentum was necrotized, it was decided to euthanize the animal.

**Figure 1:**
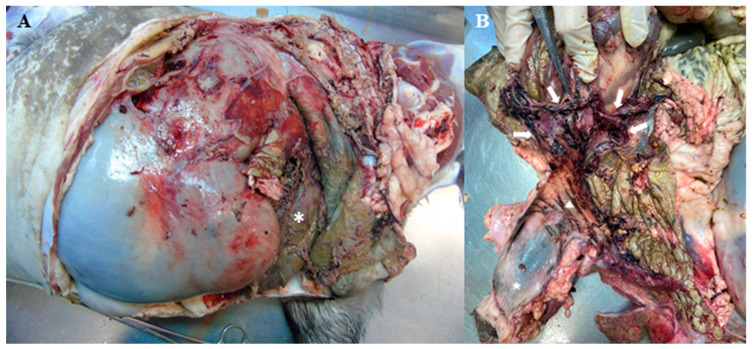
(**A**) Post-mortem examination of the abdominal cavity of a goat with septic peritonitis. The abdominal cavity shows fibrin and fragments of necrotic tissue with a brownish-green color (asterisk). (**B**) Remaining portion of the uterine body, exhibiting vulva (star), cervix (triangle), and locally extensive rupture and irregular borders (arrows).

At necropsy examination, in the abdominal cavity, there was deposition of a marked amount of fibrin and fragments of necrotized tissue in many organs (Figure 1A). The serosa of the dorsal sac and reticulum were thickened, and rough with fibrinous adherence to the left dorsal and lateral portion of the peritoneum. The ovaries and uterine horns were absent, due to ovarian-salpingo-hysterectomy; however, there was a remaining portion of the uterine body, exhibiting locally extensive rupture and irregular borders (Figure 1B). The uterine mucosa was blackish-purple and friable. There was a thick fibrous membrane covering the colon serosa and the adjacent peritoneum.

The colon segments exhibited a diffusely blackish-purple, thickened, and rough serosa and there was no sign of intestinal rupture. The liver had diffuse adherence of the diaphragmatic surface to the diaphragm. The kidneys had a surface marked by multifocal to coalescing yellowish-white spots. The rumen was filled with a moderate amount of green liquid. In the abdominal muscles, close to the rumen wall, multiple cavitary areas were filled with fibrillar material and a moderate amount of serous fluid was observed.

In the thoracic cavity, there was 6 mL of serosanguineous fluid and the lung was dark red and turgid, with only the edges of the lung lobes pink. At the opening of the pericardial sac, there was approximately 5 mL of yellow, serous fluid. It was concluded that the condition of fibrino-necrotic peritonitis was secondary to uterine rupture and there was a release of uterine contents into the abdominal cavity. Liquid rumen content and serous fat atrophy are potentially secondary to anorexia and, consequently, negative catabolism.

## 3. Discussion

In this paper, the authors reported a case of septic peritonitis secondary to uterine rupture after dystocia in a miniature goat. Small-breeds of small ruminants are more predisposed to periparturient emergences, probably because of the diameter of the pelvis relative to kid size [1]. And among the secondary complications of dystocia in small ruminants, uterine rupture is a frequent one. Both dystocia in a small-breed goat and uterine rupture after dystocia were observed in this case, including a referral of a fetus stillborn on the first day of clinical care.

The psychopathological aspects that can lead to uterine tears are not clarified. It is assumed that uterine inflammation may be a risk factor for rupture [12] once it turns the organ more friable and more prone to tears [2]. In this case, diagnostic findings compatible with metritis, such as purulent vaginal discharge and morphological uterine alterations observed during ultrasonographic examination, suggested a prior uterine inflammation process. Besides dystocia, a layperson intervention during periparturient emergence is also a risk factor for uterine rupture in small-breed goats [1]. But, according to a report from the person responsible for the goat, this kind of intervention did not occur in this case.

Unfortunately, the etiologic agent of metritis was not identified. But the most likely scenario is that the septic content of the uterus spread in the abdominal cavity, which consequently led to septic peritonitis caused by the same bacteria that caused the infectious process in the uterus.

Hematologic findings, mainly white blood cell values, are important in predicting cases of peritonitis in small ruminants [13] and were essential to monitoring inflammation due to infectious processes in this case (Table 1). Left shift leukocytosis with increased neutrophiles in the first attendance (Day 1) was remedied in the first moment but was identified again after six days (Day 7). This recurrent leukocytosis was concomitant to free fluid and peritoneum thickening observed in the ultrasonographic exam on Day 9, which indicates that peritonitis emerged or intensified during this period. The anemia reported from Day 5 onwards was likely caused by hemorrhage due to uterine rupture and the red blood cell values were just in the standard parameters after blood transfusion on Day 12 and Day 14.

Bacterial identification was performed by MALDI-TOF and antimicrobial susceptibility testing was performed by the disk-diffusion test. *E. faecium* is a clinically important pathogen from mammals’ microbiota and is often related to infections in domestic animals, and *E. casseliflavus* is mostly related to intestinal microbiota and frequently isolated from animals' fecal samples [14]. *Enterococcus* spp. have intrinsic resistance to cephalosporins, aminoglycosides, clindamycin, trimethoprim, sulfamethoxazole-trimethoprim, and fusidic acid [7]. Therefore, when an eventual infection caused by these bacteria genera is not properly identified and their antimicrobial susceptibility verified, it is likely that several ineffective antimicrobial therapies are performed, as was observed in this case report with gentamicin, enrofloxacin, ceftiofur, and penicillin therapies.

In addition, infections caused by these pathogens represent a major threat once enterococci species have additional intrinsic antimicrobial resistance. *E. faecium* has additional intrinsic antimicrobial resistance to carbapenems, *E. casseliflavus* has additional intrinsic antimicrobial resistance to vancomycin, and both antimicrobials are used in the treatment of infections caused by multi-drug resistant bacteria [7].

*Enterococcus* spp. are frequently isolated from ruminants’ vaginal microbiota [3], while there is a lack of description of this opportunistic pathogen in the uterine microbiota. In this case, the ascending migration of both enterococci species from the vagina to the uterus likely occurred, which caused metritis and spread to the abdominal cavity after uterine rupture. Cases of bacterial ascending migration, either from the vagina or from the environment through the vagina to the uterus, are common in cases of metritis [15]. The possibility of fecal contamination during the parturition cannot be discarded, considering that both bacteria species are also from intestinal microbiota [14] and the cervix was open postpartum. But, as observed in the post-mortem examination, there was no intestinal rupture; therefore, contamination by intestinal content in the abdominal cavity is improbable.

The type of treatment for uterine rupture depends on the extension of the tear. Uterine sutures can be performed, but laparohysterectomy is chosen when the organ is compromised [16,17]. A similar case with a small-breed goat was reported but the laparohysterectomy was successful since metritis and peritonitis were not observed. Once, in this case, there was omentum necrosis and adherence in many abdominal organs, the animal was euthanized. Such adherences and necrosis were confirmed in a post-mortem examination.

## 4. Conclusions

In summary, the authors present a case report of septic peritonitis caused by *E. faecium* and *E. casseliflavus* secondary to a uterine rupture in a miniature goat. To the author’s knowledge, this is the first case report of septic peritonitis caused by two different bacterial species in a small ruminant. This case shows the importance of prompt emergency attendance, obstetrical and microbiological diagnosis, and assessment of the antimicrobial susceptibility profile, aiming to make the treatment more effective and ensure the patient’s survival.

## Figures and Tables

**Table 1:** Hematology and serum biochemistry examination.

Parameters	Day 1	Day 2	Day 5	Day 7	Day 9	Day 11	Day 13	Day 14	Reference Interval [9,10,11]
RBC cell × 10^12^/L (cell × 10^6^/µL)	20.7 (20.7)	20.5 (20.5)	12.5 (12.5)	9.55 (9.55)	7.8 (7.8)	5.2 (5.2)	-	-	8–18 (8–18)
Hemoglobin g/L (g/dL)	102 (10.2)	103 (10.3)	78 (7.8)	62 (6.2)	46 (4.6)	31 (3.1)	-	-	80–120 (8–12)
HCT L/L (%)	0.3 (30)	0.3 (30)	0.24 (24)	0.18 (18)	0.14 (14)	0.09 (9)	0.11 (11)	0.21 (21)	0.22–0.38 (22–38)
MCV fL (μm^3^)	14 (14)	15 (15)	19 (19)	19 (19)	18 (18)	17 (17)	-	-	16–25 (16–25)
MCH pg/cell	4.9	5	6.3	6	6	6	-	-	5.2–8.0
MCHC g/L (g/dL)	340 (34)	340 (34)	320 (32)	340 (34)	330 (33)	340 (34)	-	-	300–360 (30–36)
Reticulocytes cell × 10^9^/µL (cell × 10^3^/µL)	-	-	-	-	-	30.3 (30.3)	-	-	-
WBC cell × 10^9^/L (cell/µL)	28 (28,000)	10.2 (10,200)	11.2 (11,200)	14.7 (14,700)	31 (31,000)	33 (33,000)	-	-	4–13 (4000–13,000)
Band neutrophils cell × 10^9^/L (cell/µL)	0	0.306 (306)	0	0.147 (147)	0.31 (310)	0	-	-	Rare
Segmented neutrophils cell × 10^9^/L (cell/µL)	23.24 (23,240)	3.162 (3162)	6.16 (6160)	7.056 (7056)	22.94 (22,940)	24.09 (24,090)	-	-	1.2–7.2 (1200–7200)
Lymphocytes cell × 10^9^/L (cell/µL)	3.38 (3380)	5.508 (5508)	4.368 (4368)	5.145 (5145)	6.2 (6200)	7.92 (7920)	-	-	2–9 (2000–9000)
Monocytes cell × 10^9^/L (cell/µL)	0.280 (280)	1.122 (1122)	0.672 (672)	2.352 (2352)	1.550 (1550)	0.990 (990)	-	-	0–0.55 (0–550)
Eosinophils cell × 10^9^/L (cell/µL)	0	0.102 (102)	0	0	0	0	-	-	0.050–0.650 (50–650)
Basophils cell × 10^9^/L (cell/µL)	0	0	0	0	0	0	-	-	0–120 (0–120)
Thrombocytes cell × 10^9^/L (cell × 10^3^/µL)	0.641 (0.641)	0.202 (0.202)	0.066 (0.066	0.227 (0.227)	0.464 (0.464)	-	-	-	0.3–0.6 (0.3–0.6)
Total protein g/L (g/dL)	57.9 (5.79)	48.2 (4.82)	44 (4.4)	-	50.9 (5.09)	57.8 (5.78)	62(6.2)	62 (6.2)	64–70 (6.4–7.0)
Albumin g/L (g/dL)	25.9 (2.59)	20.3 (2.03)	15.6 (1.56)	-	15.4 (1.54)	17.2 (1.72)	-	-	27–39 (2.7–3.9)
AST µkat/L (units/L)	0.77 (46)	2.01 (120.1)	6.97 (417.5)	-	7.27 (435.1)	5.45 (326.5)	-	-	2.79–8.57 (167–513)
GGT µkat/L (units/L)	0.25 (14.9)	0.18 (10.6)	0.08 (4.9)	-	0.39 (23.2)	0.51 (30.4)	-	-	0.33–0.94 (20–56)
Total bilirubin μmol/L (mg/dL)	7.011 (0.41)	2.736 (0.16)	1.026 (0.06)	-	4.788 (0.28)	8.208 (0.48)	-	-	0–1.71 (0–0.1)
BUN mmol/L (mg/dL)	29.881 (83.7)	39.091 (109.5)	24.704 (69.2)	-	37.164 (104.1)	20.599 (57.7)	-	-	3.57–7.14 (10–20)
Creatinine μmol/L (mg/dL)	167.076 (1.89)	174.148 (1.97)	125.528 (1.42)	-	115.804 (1.31)	116.688 (1.32)	-	-	88.4–159.12 (1.0–1.8)

Abbreviations: AST = aspartate aminotransferase; BUN = blood urea nitrogen; GGT = gamma-glutamyltransferase; HCT = hematocrit; MCH = mean corpuscular hemoglobin; MCHC = mean corpuscular hemoglobin concentration; MCV = mean corpuscular volume; RBC = red blood cell; WBC = white blood cell.

**Table 2:** Antimicrobial susceptibility profile of Enterococcus faecium and Enterococcus casseliflavus isolated from peritonitis fluid of a goat.

	Susceptibility Profile [7]	
Antimicrobial Tested	*Enterococcus faecium*	*Enterococcus casseliflavus*
Ampicillin	Resistant	Sensible
Penicillin	Resistant	Sensible
Chloramphenicol	Sensible	Intermediate
Ciprofloxacin	Resistant	Intermediate
Erythromycin	Intermediate	Intermediate
Tetracycline	Resistant	Sensible
Vancomycin	Sensible	Intermediate

## Data Availability

The data presented in this study are available in the article.
